# Deoxyarbutin Possesses a Potent Skin-Lightening Capacity with No Discernible Cytotoxicity against Melanosomes

**DOI:** 10.1371/journal.pone.0165338

**Published:** 2016-10-24

**Authors:** Fang Miao, Ying Shi, Zhi-Feng Fan, Shan Jiang, Shi-Zheng Xu, Tie-Chi Lei

**Affiliations:** Department of Dermatology, Renmin Hospital of Wuhan University, Wuhan, China; Wenzhou Medical University, CHINA

## Abstract

Safe and effective ingredients capable of removing undesired hyperpigmentation from facial skin are urgently needed for both pharmaceutical and cosmetic purposes. Deoxyarbutin (4-[(tetrahydro-2H-pyran-2-yl) oxy] phenol, D-Arb) is a glucoside derivative of hydroquinone. Here, we investigated the toxicity and efficacy of D-Arb at the sub-cellular level (directly on melanosomes) and skin pigmentation using *in vivo* and *in vitro* models to compare with its parent compound hydroquinone (1,4-benzenediol, HQ). At first, we examined the ultrastructural changes of melanosomes in hyperpigmented guinea pig skin induced by 308-nm monochromatic excimer lightand/or treated with HQ and D-Arb using transmission electron microscopy. The results showed that prominent changes in the melanosomal membrane, such as bulb-like structure and even complete rupture of the outer membranes, were found in the skin after topical application of 5% HQ for 10 days. These changes were barely observed in the skin treated with D-Arb. To further clarify whether membrane toxicity of HQ was a direct result of the compound treatment, we also examinedultrastructural changes of individual melanosomes purified from MNT1 human melanoma cells. Similar observations were obtained from the naked melanosome model *in vitro*. Finally, we determined the effects of melanosomal fractions exposed to HQ or D-Arb on hydroxyl radical generation in the Fenton reaction utilizing an electron spin resonance assay. D-Arb-treated melanosomesexhibit a moderate hydroxyl radical-scavenging activity, whereas HQ-treated melanosomessignificantly generate more hydroxyl free radicals. This study suggests that D-Arb possesses a potent ability in skin lightening and antioxidation with less melanosome cytotoxicity.

## Introduction

Melasma (chloasma) is a common skin pigmentary disorder characterized by irregular light to darkbrown patches on the face, which usually cause significant psychiatric and psychological burdens for affected individuals. However, the inciting events for the pathogenesis of melasma are not well understood [1, 2]. Melasma is more common in persons with Fitzpatrick skin types IV through VI than in those with fairer skin (types I through III). Melasma is also recognized to be more common among women than men and more common among Asians, Latinos and African-Americans than among Caucasians. An estimated 50% to 70% of pregnant women in the US develop melasma [3]. It has been established that the hyperactive melanocytes in melasma skin produce much more melanin pigment via the tyrosine catabolism pathway [4]. Tyrosinase (EC 1.14.18.1), a copper-containing glycoprotein, is the rate-limiting enzyme critical for this pathway. Melanin synthesis occurs within highly specialized organelles termed melanosomes and then melanized melanosomes are transferred from melanocytes to neighboring keratinocytes, thereby determining constitutive and facultative skin color. Many skin lightening agents are targeted on controlling melanin synthesis via the suppression of tyrosinase activity [5]. Over the past50years, hydroquinone (1, 4-benzenediol, HQ) has been widely used as the gold standard for prototypical tyrosinase inhibitors in treating melasma[5–6]. However, much recent attention has been paid to the potential health risk of long term exposure to HQ, such as exogenous ochronosis, leukoderma (occupational vitiligo), and even carcinogenesis [7–8].It is generally accepted that HQ presumably acts as a substrate to competitively inhibit tyrosinase activity, granting the potential of skin whitening. Meanwhile, these monophenol compounds also give rise, through the enzymatic oxidation by tyrosinase, to abundant free radicals that cause lipid peroxidation and consequent melanosomal membrane damages [9–10].In previous studies from our laboratory it has been shown that deoxyarbutin (4-[(tetrahydro-2H-pyran-2-yl) oxy] phenol, D-Arb), a glucoside derivative of HQ, is safer and less cytotoxic when compared to its mother compound hydroquinone [11]. The present study was undertaken to investigate the toxicity and efficacy of D-Arb at the sub-cellular level (directly on melanosomes) and skin pigmentation using *in vivo* and *in vitro* models, further evaluating the biosafety of D-Arb in parallel comparison with HQ for skin lightening use.

## Materials and Methods

### 1. Monochromatic excimer light (MEL) irradiationto induce hyperpigmentation in brownish guinea pigs

Five-week-old male brown guinea pigs (weight 400-450g each) were purchased from theexperimental animal facility of Wuhan University (Wuhan, China).The experimental protocol for this study was approved by the InstitutionalAnimal Care and Use Committee at the Renmin Hospital of Wuhan University. The guinea pigs were housed in a temperatureand humidity-controlled room (23±1°C, 50±5% humidity) with a 12 h light/dark cycle. After 1 week of quarantine, the guinea pigs were acclimated to individual cages. During the experimental period, food and water were given *ad libitum*.The high energy MEL source was supplied by a 308-nm excimer lamp delivery system (USHIO, Tokyo, Japan). MEL irradiation was applied to the dorsal skin of each guinea pig after hair removal.Each irradiation dose was 125 mJ/cm^2^, every other day for a total of four exposures. The total irradiation dose of 500 mJ/cm^2^ was given to the dorsal skin in 6 separate rectangular irradiating areas of 1.5×1.5 cm^2^. Each experiment included three guinea pigs from at least two different litters. All animals were euthanized via CO_2_ inhalationat the end of the experiment.

### 2. Chemicals and treatments

**Fig 1:**
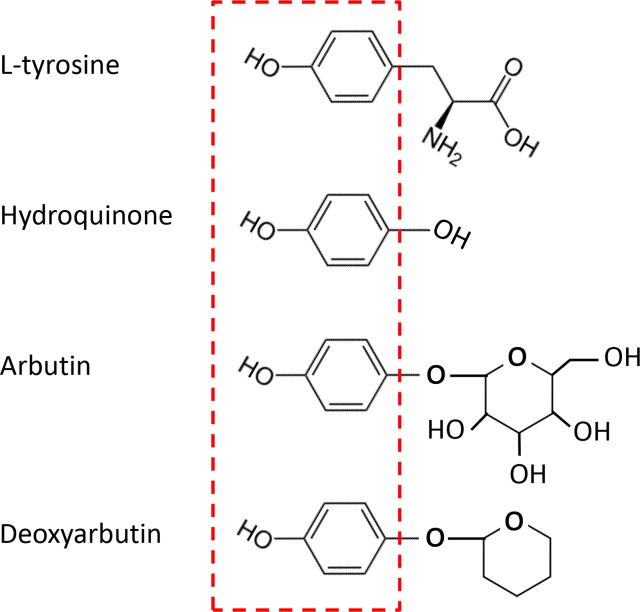
Chemical structures of tyrosine, HQ, arbutin and D-Arb. HQ, arbutin, and D-Arb share a core phenolic moiety with tyrosine in their chemical structures, as shown in red broken-line box.

HQ, arbutin and hydrogen peroxide (H_2_O_2_) were purchased from Sigma–Aldrich Corp. (St.Louis,MO, USA). D-Arb was synthesized by Dr. Sheng-Feng Ding accordingto a previous report [11]. Fig 1 denotes that HQ, arbutin, and D-Arb share a core phenolicmoiety with tyrosine in their chemical structures.All compoundswere dissolved inDMSO and stock solutionsof compounds were prepared and protected from light at -20°Cuntil used. At the time of use, the compounds were further diluted in growth medium to the final concentration. For the animal study, 5% HQ, 10% arbtuin, and 10% D-Arb were prepared in a mixture of propylene glycol, ethanol, and water at a volume ratio of 1:2:1. The oil-in-water (O/W) cream base used in this study was supplied bythe Department of Pharmaceutics, Renmin Hospital of Wuhan University. Topical preparation of hydroquinone was extremely unstable and rapidly discolors in appearance owing to oxidation. All creams containing a testing compoundwere freshly made and only used within 1 hour. The 3% H_2_O_2_ solution and freshly prepared cream were applied topically to the dorsal skin once a day for 10 days after the final 308-nm MEL irradiation.The cream base only was used as a vehicle control. The skin color in topically-treated areas was recorded using a CR100 Minolta chromameter (Konica Minolta Sensing Inc, Tokyo, Japan) and a digital camera. Color changes were measured numerically by the CIE L*a*b* system.

### 3. Melanin content and distribution in skin visualized by Fontana-Masson staining

Fontana–Masson staining was carried out to visualize melanin content and distribution in irradiated dorsal skin as described previously [12]. Sections were deparaffinized and hydrated in water and placedinto a 2.5% silver nitrate solution (Sinopharm Chemical Reagent Co., Ltd, Beijing, China) for 4 h in a dark chamber. After rinsing in water, the slides were placed in 0.1% gold chloride (Sinopharm Chemical Reagent Co., Ltd, Beijing, China) for 1 min,rinsed again in water, and then in 5% sodium thiosulfate (Sinopharm Chemical Reagent Co., Ltd, Beijing, China) for an additional2 min. After a final rinse with water, the sections were counter-stained with eosin red.

### 4. Melanin content and distribution in the surface corneocyte visualized by Fontana-Masson staining

Melanin content and distribution in the corneocyte was estimated using a surface strippingslide mounting technique as described previously [13]. Surface corneocytes were sampled by pressing a piece of clear adhesivetape (Scotch, 3M Company, St. Paul, MN, USA) on the skin, which was then removed andtransferred to slides that had been thinly coated with a vinyl chloride resin. The slides were immersed in xylene for several hours until all remaining adhesivematerials were dissolved, leaving a layer of surface corneocytes on the slide. After evaporation of xylene,the slides were stained overnight at room temperaturewith Fontana-Masson ammoniac silver nitrate, andthen slightly stained with haematoxylin and eosin. Microscope images were captured using a 100× objective lens. The number of black silver particles was counted in isolated corneocytes of 10 randomly selected fieldsacross each specimen.

### 5. Cell line and cell culture

MNT-1 human melanoma cells, in which mature (stage III and IV) melanosomes are highly enriched, were generously provided by Dr. Vince Hearing (Pigment Cell Biology Section, NCI, NIH, USA). Briefly, these cells were grown in DMEM (Gibco, Life Technologies, Rockville, MDUSA) supplemented with 10% fetal bovine serum (Hyclone, Logan, UT, USA), 100 U/mL penicillin G, 100 μg/mL streptomycin sulfate and 2 mM glutamine [14]. All cells were maintained at 37°C, 95% humidity and 5% CO_2_.

### 6. Preparation and treatment ofmelanosome-enriched subcellular fractions

We used a previously described protocol to isolate the individual naked melanosome-rich fraction [15–16]. Briefly, confluent monolayers of MNT-1 cells were harvested and kept frozen at 2×10^6^cells per Eppendorf tube (1.5 mL). After thawing the frozen cells, 1 mL cold lysis buffer, consisting of 0.1 M Tris-HCl, pH 7.5, 1% Igepal CA-630 (Sigma-Aldrich, I3021) and 0.01% SDS, was added to the cells and was stored at 4°C for 20 min with mixing every 10 min. After centrifugation (1×10^3^gfor 5 min at 4°C), thesupernatants were transferred to new Eppendorf tubes (1.5 mL) and centrifuged again in the same manner. The supernatants were then further centrifuged (2×10^4^gfor 5 min at 4°C) and the precipitates were washed twice with D-PBS and brief and gentle mixing in order to avoid dispersion and were then centrifuged again (2×10^4^g for 5 min at 4°C). The pellets were then used immediately as the individual melanosome-enriched fraction. For the treatment of melanosome specimens with various types of oxidative stress, the naked melanosomes were resuspended in PBS and then exposed to 3J/cm^2^ UVA radiation from a bank of 9 Philips UVA lamps (320-380nm) with a peak emission at 350nm (Sigma High-Tech Co., Ltd, Shanghai, China). Furthermore, the melanosomes were incubated with fresh medium containing 10 μM HQ, 100 μM D-Arb or 100 μM H_2_O_2_ at 37°C for 30 min. It was worth mentioning that no inhibition of 10 μM HQ, 100 μM D-Arb or 100 μMH_2_O_2_on cell viabilities of cultured melanocytes was observed according to our previous study [11]. The melanosomes were also treated using the physical approach of multiple freeze-thaw cycles and manual grinding using a glass Dounce homogenizer (Wheaton, Millville, NJ, USA) and a chemical method of solubilization in 8M urea (Sigma-Aldrich Corp., St. Louis, MO, USA), used as a positive control for melanosome breakdown.

### 7. Ultrastructural observation of melanosomes by Transmission Electron Microscopy (TEM)

Skin samples and melanosome pelletswere harvested and fixed with 2% glutaraldehyde in D-PBS at 4°C for at least 2 h. After washing twice with D-PBS for 15 min each, the cells were post-fixed with2% osmium tetroxide for 1.5 h. After fixation, they were dehydrated in a graded series of ethanol and propylene oxide, and embedded in epoxy resin (EPON) for 48 h at 60°C. Ultrathin sections were cut and then stained with uranyl acetate and lead citrate and were examined using atransmission electron microscope (JEM-1200EX, JEOL, Japan) at 100 kV. At least five TEM images were randomly taken from each melanosome specimen. The percentage of damaged melanosomes was calculatedto represent the efficacy of melanosomal degradation.

### 8. Fenton reaction and hydroxyl radical measurement by electron spin resonance (ESR) spin trapping assay

All solutions except FeSO_4_ were dissolved in 0.1 M phosphate buffer (pH 7.4); FeSO_4_ was dissolved in distilled water. The effects of untreated/treated melanosome samples on hydroxyl radical (•OH) generation in the Fenton reaction was analyzed using a spin trapping ESR method, as reported previously [12, 17]. Each reaction was carried out in 50 μl aqueous solution, in an Eppendorf tube containing 260 mM H_2_O_2_, 0.4 mMFeSO_4_, 400 mM spin trapping reagent (DMPO) and melanosome samples mentioned above.In the control, metal-free water was substituted for the sample. The Fenton reaction was initiated by the addition of H_2_O_2_, then 50 μl of the reaction mixture was placed in an ESR quartz flat cell. Exactly 20s after the addition of H_2_O_2_, the ESR spectra of the DMPO-OH spin adducts were recorded at room temperature using a Bruker ER 200D-SRC ESR spectrometer (Bruker Analytische Messtechnik GmbH, Rheinstetten, Germany) operating at 9.53 GHz microwave frequency, 20 mW microwave power, 100 kHz modulation frequency and 0.05 mT modulation amplitude.

### 9. Statistical analyses

Data are expressed as mean ± SD from at least 3 independent experiments. Statistical analyses were performed using GraphPad software (San Diego, CA, USA). Statistical differences among groups were determined using two-way analysis of variance (ANOVA). *P* values<0.05 are considered significant.

## Results

### 1. Potency of D-Arb and HQ on skin lightening and effects on melanosomal ultrastructure in hyperpigmented guinea pig skin

**Fig 2:**
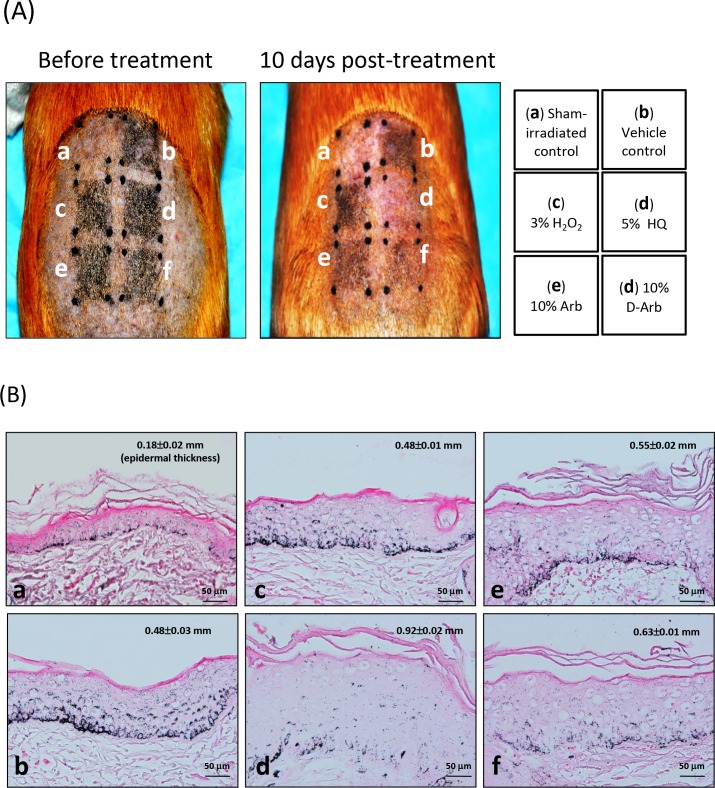
Phenotypic changes and melanin distribution in hyperpigmented guinea pig skin treated with HQ. A: Hyperpigmentation in guinea pig dorsal skin was induced by 308-nm MEL radiation. Changes in skin pigmentation were observed at the irradiated sites treated with (c) 3% H_2_O_2_, (d) 5% HQ, (e) 10% arbutin or (f) 10% D-Arb, once a day for 10 days, as described under Materials and methods. (a) Sham-irradiated control and (b) vehicle control. B: Fontana-Masson staining reveals supranuclear melanin as black-brown granules in the basal and suprabasal layers in skins of the vehicle control (b). Less pronounced melanin deposits are seen in (d) 5% HQ or (f) 10% D-Arb. It is noted that HQ caused an increase in epidermal thickness (d). Scale bar, 50 μm.

**Fig 3:**
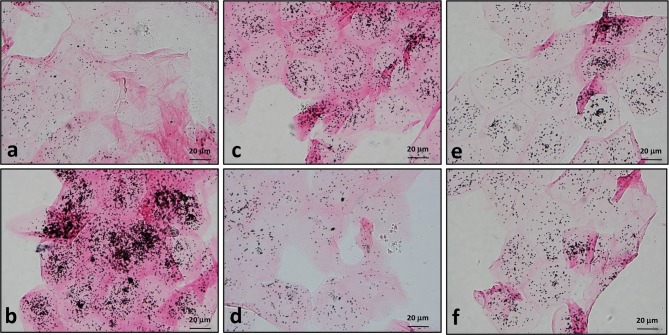
Measurement of melanin remnants in surface corneocytes using Fontana-Masson silver staining. Surface corneocytes were collected from guinea pig dorsal skin treated as described in Fig 2. Many coarseclustered black silver deposits are seen in corneocytes from vehicle controls (b)and H_2_O_2_-treated group (c), whereas fewer silver precipitations are seen in the sham-irradiated control (a) andthe depigmented skin after topical application of 5% HQ (d), 10% arbutin (e) and 10% D-Arb (f) for 10 days. Scale bars: 20 μm.

**Fig 4:**
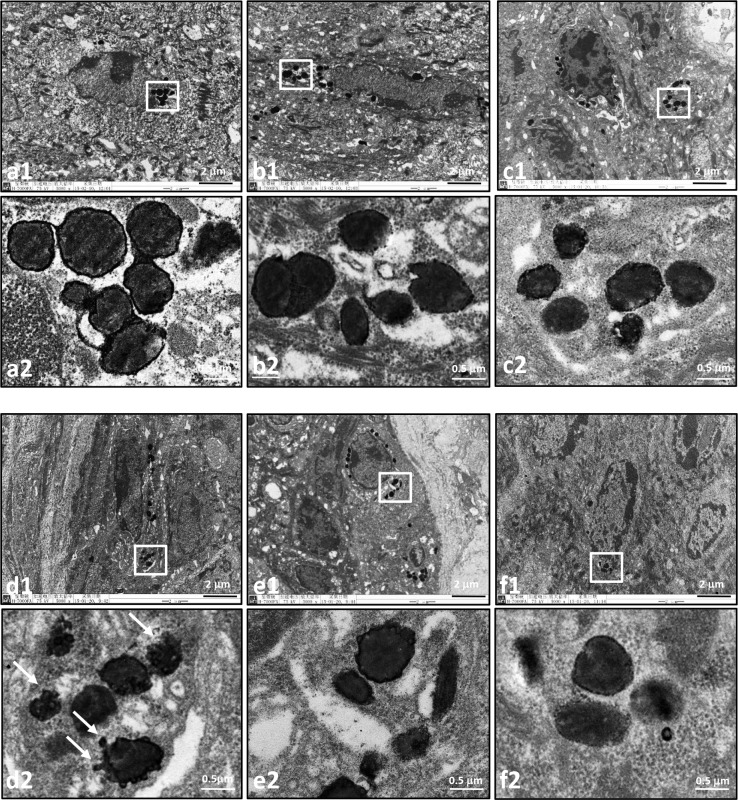
Ultrastructural observation of melanosomes within keratinocytes of hyperpigmented guinea pig skin treated with HQ. The dorsal skins of guinea pigs were treated as described for Fig 2. Marked vacuolations and fragmentations of melanosomes are seen in the depigmented skin after topical application of 5% HQ for 10 days (d1 and d2, arrows). The outer membranes of melanosomes in skins from (a) sham-irradiated control, (b) vehicle control, and (f) 10% D-Arb seem to be intact or have minor damage. Low-magnification views (a1-f1)are shown in the upper panels, the white boxes mark the areas magnified in the panels below (a2-f2). Scale bars: 2 μm(low-); 0.5 μm (high-magnification).

The shaved dorsal skins of brownish guinea pigs were repeatedly exposed to MEL irradiation to achieve hyperpigmentation. Subsequently, the irradiated sites were given 10 days of topical treatment with cream base (b, vehicle control), 3% H_2_O_2_ (c), 5% HQ (d), 10% arbutin (e) or 10% D-Arb (f), as indicated in Fig 2A. The site covered with an aluminum foil served as a shamirradiated control (a). The CIE-L* values werethen measured to represent skin brightness, as shown in Table 1. The L*value was significantly higher in the HQ-treated group (51.50±0.4) than in the vehicle control group (47.3±0.5) (*P*<0.05). A similar effect of skin bleaching was seen in the D-Arb-treated group (50.9±0.4) compared with the HQ-treated group (*P*>0.05). Sections of each tissue were subjected to Fontana-Masson silver staining, which allows the visualization of melanin content and distribution in the epidermis. Figs 2 and 3 show that large amounts of silver-stainedmelanin particles were distributed throughoutthe epidermis or the surface corneocytes in the mock control group. D-Arb exerted a more powerful action on reducing the number of these melanin granules from the irradiated skins with comparable results to HQ. To further characterize whether both compounds induce melanosome membrane damage *in vivo*, the skin tissues were examined using TEM. The ultrastructural changes of melanosomes are shown in Fig 4. Most of the outer membranes of melanosomes remained intact in the vehicle control group. In sharp contrast to this, marked ruptures in melanosomal membranes were found in the depigmented skin after topical application of 5% HQ for 10 days. Some melanosomes occasionally manifested a bulb-like structure in the outer membranes, as shown in Fig 4D. A discernible damage on melanosomal membrane was barely observed in the skin treated with D-Arb. The percentage of damaged melanosomes was significantly higher in the HQ-treated group (17±2.5%) than in D-Arb-treated group (2.3±0.4%) (*P*<0.05, Table 1).

**Table 1:** Comparisons of epidermis thickness, L* value, damagedmelanosomes and melanin particles in skins treated with H_2_O_2_, HQor its derivatives.

Group	L* value	Epidermis thickness (mm)	Damaged MS(%)	Melanin particles (per corneocyte)
Mock control	52.7±0.58	0.18±0.02	1.6±0.2	31.7±10.2
Vehicle control	47.3±0.5	0.48±0.03	1.8±0.4	190.0±26.5
3% H_2_O_2_	46.9±0.1	0.48±0.01	4.8±1.1	58.3±16.1
5% HQ	51.5±0.4*	0.92±0.02	17.1±2.5*	33.7±5.5*
10% Arb	49.1±0.5	0.55±0.02	6.7±2.1	57.0±11.2
10% D-Arb	50.9±0.4	0.63±0.01	2.3±0.4	40.2±9.5

L*values were measured to represent skin brightness at all test sites of the 3 guinea pigs; The thickness of epidermis in each field was measured at all test sites under microscopy; The percentage of damaged melanosomes was calculated from 5 TEM images taken from each specimen. Data represent the mean ± SD of three independent experiments.

*P <0.05 compared to vehicle control group by two-way ANOVA.

### 2. Effects of D-Arb and HQ on the ultrastructure and function of individual naked melanosomes *in vitro*

**Fig 5:**
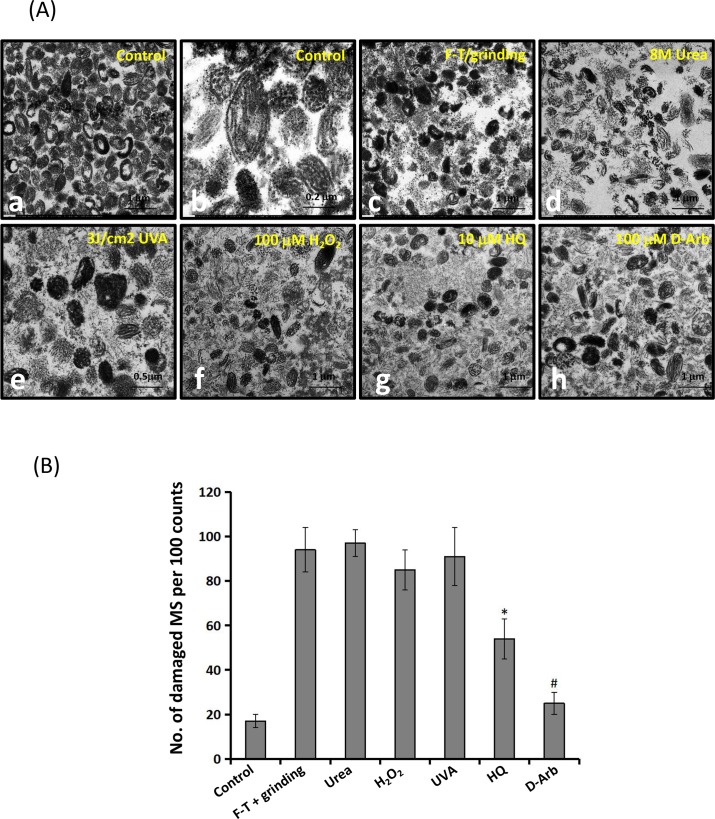
Ultrastructural observations of individual naked melanosomes. A: Individual naked melanosomes were purified from cultured MNT1 cells, as described under Materials and methods. Typical images of mature melanosomes are shown in low (a) (5000×) and in higher (b) (15000×) magnifications. Melanosome fractions were treated with freeze-thawing (FT) plus manual grinding (c), 8M urea (d), 100 μM H_2_O_2_ (e), 3J/cm^2^ UVA radiation(f), 10 μM HQ (g) and 100 μM D-Arb (h). Significantly fragmented and vacuolated melanosomes are seen in specimens treated with 100 μM H_2_O_2_, 3 J/cm^2^ UVA radiation, and 10 μM HQ (e-g). Scale bar: 1μm (except 0.2 μm for b and 0.5 μm for e). B: Comparison of percentages ofdamaged melanosomes following the different treatments. Two-way ANOVA was used to determine the statistical difference between treated melanosomes and the untreated control. * *P*<0.05, ^#^ *P*> 0.05.

To confirm the level of cytotoxicity of both compounds on melanosomes, *in vitro* studies using individual melanosomes as a model were carried out to distinguish the ultrastructural changes that ensue after different treatments, rather than from other causes. Fig 5 shows that 100 μM H_2_O_2_ (e), 3 J/cm^2^ UVA radiation (f), and 10 μM HQ (g) cause severe destruction of melanosomal membranes compared with the controls, but negligible damage is seen in 100 μM D-Arb-treated melanosomes (h). The percentage of damaged melanosomes in the 10 μM HQ-treated group was similar to that in the 100 μM H_2_O_2_ group (*P*>0.05). Moreover, we also compared the efficacy of melanosome degradation by different manners. Also noted in Fig 5C that the physical method, a combination of multiple freeze-thaw(FT) cycles with manual grinding, broke melanosomes down into several large pieces, but only minor damage was seen in their outer membranes. The chemical approach (8M urea) seemed to perform better to extract all melanogenic proteins from stage IV melanosomes to allow their intralumenal fibrillar matrix to become visible (Fig 5D), leading to the severe destruction of melanosomal membranes. However, the oxidative stress-based treatments, such as 3 J/cm^2^ UVA radiation and 100 μM H_2_O_2_ primarily destroyed the outer membrane structures in melanosomes. Ultrastructural changes of melanosomes exposed to HQ are similar to those induced by the oxidative stress.

### 3. Effects of D-Arb and HQ on the pro-oxidative activity of melanosomal fractions

**Fig 6:**
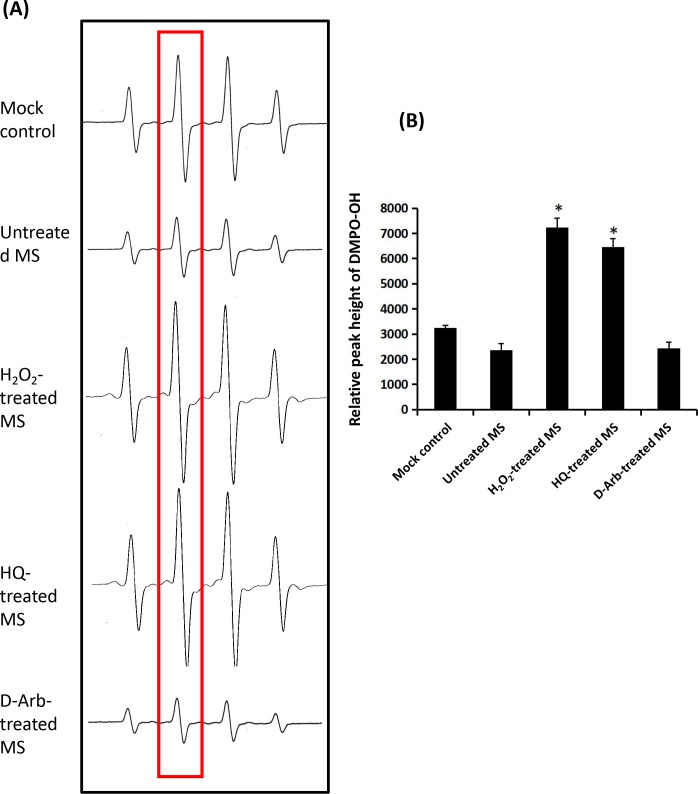
Effects of melanosomes treated with HQ on hydroxyl radical generation in theFenton reaction. A: Representative ESR spectra of DMPO–OH with melanosomes treated with 100 μM H_2_O_2_, 10 μM HQ or 100 μM D-Arb. Hydroxyl radicals are generated by the Fenton reaction (DMPO: 400 mM). B: Comparison of hydroxyl radical-scavenging activity for melanosomes with different treatments. Two-way ANOVA was used to determine the statistical difference between treated melanosomes and the untreated control. * *P*<0.05.

We hypothesized that the treatment of isolated melanosomes with or without HQ and D-Arb treatments might affect the anti-oxidant/pro-oxidant activities of melanosomes *per se*. To test that hypothesis, we determined the effects of melanosomes treated with 10 μM HQ, 100 μM D-Arb or 100 μM H_2_O_2_ on hydroxyl radical generation in theFenton reaction utilizing ESR. The results show that intact melanosomes exhibit a mild •OH scavenging activity in the Fenton reaction, whereas melanosomes treated with 10 μM HQ or with 100 μM H_2_O_2_ act as a pro-oxidantto generate much more hydroxyl free radicals thanthe mock control (Fig 6). It was surprising that 100 μM D-Arb did not display increased (•OH) ESR signals. These results indicate that the melanosome fraction exposed to D-Arb moleculesexhibits a considerable anti-oxidative activity, likely protecting melanosomal membrane from oxidative damage.

## Discussion

Increased understanding of molecular mechanisms underlying melanogenesis in recent years has driven the development of many novel therapies by which skin lightening is successfully achieved [1, 2, 18]. Laser and light therapies represent potentially promisingoptions for subjects with refractory melasma, but also carry a significant risk of worsening thedisease [19–20]. HQ still appears to be indispensable as the first-line treatment of melasma, however, many biosafety concerns have been raised in recent years in regards to the long-term use of HQ as an active ingredient supplemented in cosmetic products and daily necessities [18]. As already reported, exogenous ochronosis, irreversible skin depigmentation, even leukemia, etc. has the potential to occur in individuals who are exposed to large doses of HQ over extended time periods [21–22]. For the biosafety reason, HQ use has been forbidden in the European Union as an active ingredient in cosmetics since 2001 and a ban on over-the counter HQ was proposed by the US Food and Drug Administration (FDA) on 2006 [23]. Therefore, one safe and effective alternative to HQ for use in skin lightening is highly desirable [24–26].

Arbutin (hydroquinone-b-D-glucopyranoside) is the first glucoside derivative of HQ to be successfully used in the treatment of melasoma [27]. Subsequently, another glucoside derivative of HQ (D-Arb) has been synthesized by the removal of pendant hydroxyls from the glucose side-chain of arbutin [28–29]. More recently, Boissy and his colleagues reported that three second-generation derivatives of D-Arb, deoxyFuran (dF), 2-fluodeoxyArbutin (fdA), and thiodeoxyArbutin (tdA), also exerted potent tyrosinase inhibition, lessened cytotoxicity, and certain antioxidative potential. It is possible to serve as an effective and safe alternative to hydroquinone for use in skin lightening [30]. It is worth noting thatHQ appears to have much more cytotoxicity against melanocytes, especially in the melanosome membrane. Such cytotoxicity may result from that the rapid spontaneous oxidation or / metabolic oxidation since HQ acts as an alternate substrate to tyrosine in the intracellular tyrosinase-enriched milieu. Considering that D-Arb still has a corephenolic moiety in its chemical structure, it is imperative to investigate whether D-Arb has inherenttoxicity against melanosomessimilar to that produced by HQ. To our knowledge, no previous studies have evaluated the toxicity of HQ and D-Arb on purified melanosomes and further investigated the effect on the pro-oxidative activity of the melanosomal fractions.

In this study, we investigate the toxicity and depigmentation potential of D-Arb in a parallel comparison with those of HQ. The results show that D-Arb possesses a potent skin-lightening capacity, as shown in Figs 2 and 3, but no discernible cytotoxicity against melanosomes was found both in hyperpigmented skin induced by UVR and in individual naked melanosomes. In contrast to that, HQ severelydisrupts the outermembranestructure of melanosomes, even causes some vacuolatedand/or fragmentedchanges, as visualized in Fig 4 by TEM, which is similar to those observed in melanosomes exposed to UVA radiation or H_2_O_2_ (Figs 4 and 5). The ESR spectra (Fig 6) display significant increases in melanosomes treated with 10 μM HQ, suggesting that HQ-treated melanosome fractions exert a potentpro-oxidant activity, whereas D-Arb-treated groups exhibit a mild •OH scavenging activity in the Fenton reaction. These findings are in good agreement with the results obtained in cell level [11]. However, it is uncertain as to whether D-Arb confers a resistance against tyrosinase-mediated or spontaneous oxidation. In addition, we also note that topical application of HQ and its glucoside derivatives can enhance epidermal hyperplasia, as shown in Fig 2B. It deserves further scrutiny whether those phenol compounds accelerate pigment dilution by increasing keratinocyte proliferation or/ by epidermal turnover.

In conclusion,our findings unequivocallydemonstrate that D-Arb is a promising candidate to serve as a skin lightening ingredient with the advantages of potent tyrosinase inhibition, less cytotoxicity and even antioxidation to some extent. In the future, a controlled clinical trial in human volunteers is needed to examine the biosafety of D-Arb in different cosmetic pharmaceutical formulas [31].
